# Determining the Gibbs Energy Contributions of Ion and Electron Transfer for Proton Insertion in ϵ‐MnO_2_

**DOI:** 10.1002/cphc.202200364

**Published:** 2022-10-17

**Authors:** Keyvan Malaie, Fritz Scholz, Uwe Schröder, Harm Wulff, Heike Kahlert

**Affiliations:** ^1^ Institute of Biochemistry University of Greifswald Felix-Hausdorff-Str. 4 17487 Greifswald Germany; ^2^ Institute of Physics University of Greifswald Felix-Hausdorff-Str. 6 17489 Greifswald Germany

**Keywords:** manganese oxide, Gibbs energy, proton insertion, standard potential, electron transfer

## Abstract

Electrochemically active ϵ‐MnO_2_ and ɣ‐MnO_2_ as tunnel‐type host‐guest structures have been extensively studied by crystallography and electrochemical techniques for application in battery cathode materials. However, the Gibbs energies of the underlying ion and electron transfer processes across the electrode interfaces have not yet been determined. Here we report for the first time these data for ϵ‐MnO_2_. This was possible by measuring the mid‐peak potentials in cyclic voltammetry and the open‐circuit potentials under electrochemically reversible conditions.

## Graphical abstract

**The Gibbs energy** contributions of ion‐ and electron‐transfer steps for the proton‐insertion battery cathode ϵ‐MnO_2_ were determined for the first time based on its reversible voltammetry and open‐circuit potentiometry responses, and they were used to discuss its performance.

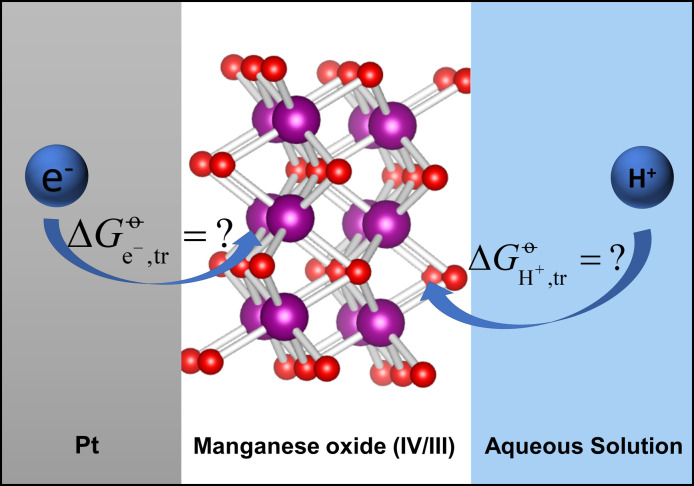

## Introduction

Manganese oxide (ɣ‐, ϵ‐, and electrolytic manganese oxide (EMD)) has been used as cathode material in commercial alkaline batteries, and it has been widely investigated as ion‐insertion cathode in non‐aqueous and aqueous electrolytes^[1]^ as well as (pseudo)capacitor in neutral electrolytes^[2]^ owing to its electrochemical activity and sustainability merits.

Despite all the interest in MnO_2_, the energetics of its underlying insertion electrochemical reactions is very poorly understood.^[3]^ There is no information on the relative contribution of ion and electron transfer processes to the overall Gibbs energy of the electrochemical reactions of MnO_2_, while this information is very important for its performance.^[4]^

Although the individual Gibbs energies of ion and electron transfer could be discerned in case of three‐phase electrodes consisting of immobilized organic solvent droplets in which a redox probe is dissolved, and ions are exchanged with the surrounding aqueous solution,[^5^, ^6^] the same could be achieved for solid insertion electrochemical systems only recently. In 2015, Scholz et al. for the first time could separate the contribution of ion and electron transfer for a tungsten bronze by comparing the reversible voltammetric and potentiometric responses of this compound.[^7^, ^8^] Later, Doménech Carbò et al. used this method to determine the individual Gibbs energies of ion and electron transfer for a tetranuclear gold complex appended to ferrocenyl units.^[9]^

Here, for the first time, we report the Gibbs energies of ion and electron transfer of a battery material, i. e., of ϵ‐MnO_2_. First, we describe the one‐electron and two‐electron electrochemical insertion reactions of MnO_2_ and their different equilibrium potentials in potentiometry and cyclic voltammetry. Then, they are experimentally verified and measured in a practically reversible system. Next, the individual Gibbs energies of ion and electron transfer for the proton insertion/deinsertion reaction are determined.

## Results and Discussion

**Scheme 1:**
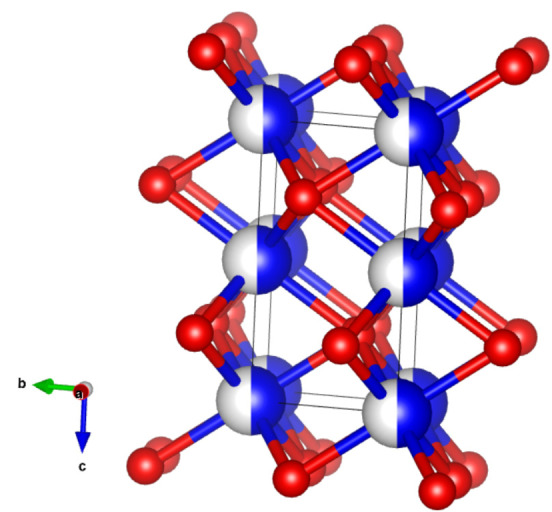
Hexagonal cell of ϵ‐MnO_2_ (space group P63/mmc) along the a‐axis and the plausible tunnels for diffusion of protons. (blue‐white: Mn or vacancy and red: O; ionic sizes are not in the correct scale).

Scheme 1 illustrates the crystal pattern of the unit cell of ϵ‐MnO_2_ (see Sec. 2.1 in Supporting Information, ESI for XRD analysis). ϵ‐MnO_2_ is a highly defective crystal in which Mn^4+^ cations (blue‐white spheres) randomly occupy only 50 % of the octahedral sites of the hexagonal‐close‐packed (hcp) oxygen sublattice. These MnO_6_ octahedra share their edges and corners to create 2×1 and 1×1 tunnels, unlike pyrolusite (β‐MnO_2_) which has only 1×1 tunnels. It is believed that proton transport in these 2×1 tunnels occurs more easily (as also in the case of ɣ‐MnO_2_ and EMD) compared to the 1×1 tunnels of pyrolusite, probably due to the larger tunnel size and the relative positions of oxygen and manganese atoms along the tunnels.[^10^, ^11^]

**Scheme 2:**
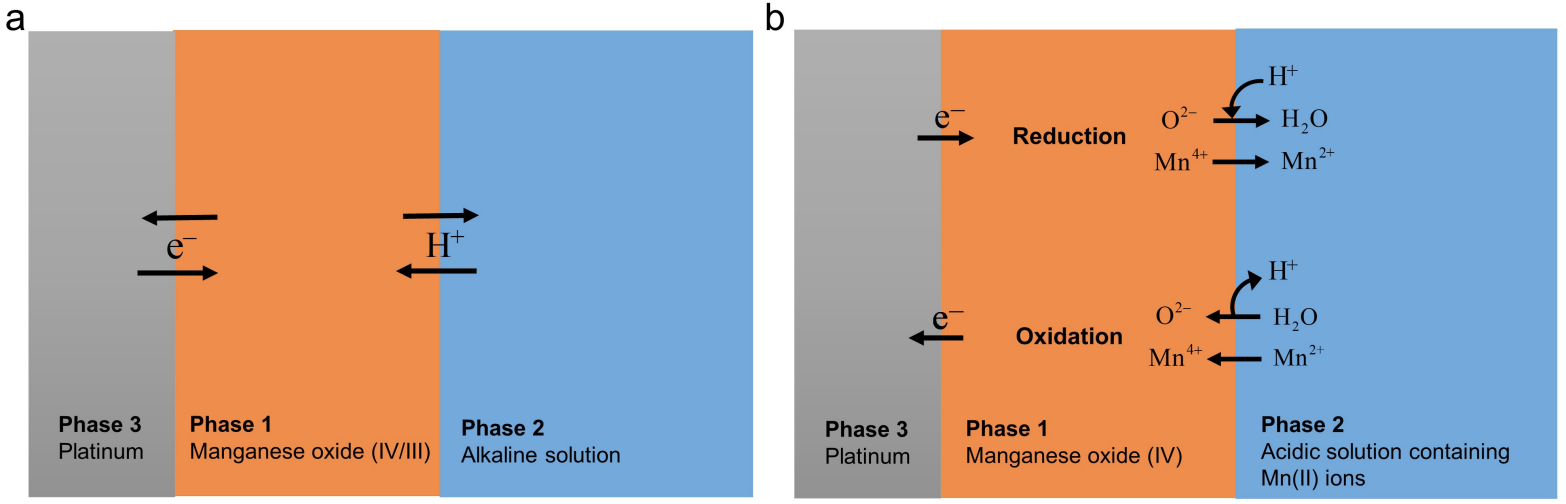
Schematic illustrations of the one‐electron, one proton‐transfer (a) and two‐electron transfer (b) reactions of MnO_2_ and their electron and ion transfer steps at two separate interfaces in conjunction with two different aqueous solutions.

Generally, there are two different electrochemical reactions of MnO_2_ in conjunction with aqueous solution: 1) a one‐electron, one proton transfer reaction between Mn(IV) and Mn(III) in alkaline electrolyte, i. e., the proton insertion/deinsertion reaction, (Eq. 1^[11]^); and 2) a two‐electron transfer reaction between Mn(IV) and Mn(II) coupled to the dissolution of the oxide in mild‐acidic electrolyte^[12]^ (Eq. 2, cf. Scheme 2):

(1) $${MnO}_{2}{+H}^{+}+e^{-}\vec{\leftarrow }MnOOH$$

(2) $${MnO}_{2}{+4H}^{+}+{2e}^{-}\vec{\leftarrow }{Mn}^{2+}+2H_{2}O$$

In OCP measurements practically no current flows in the two systems and there is only one potential‐determining interface, i. e., oxide‐solution interface, in addition to the junction potential of the reference electrode which is constant in all the measurements. When the OCP has finally reached a constant value, it is probable that the system has reached its equilibrium state. Then the ions on both sides of the interface acquire equal electrochemical potentials in analogy to ion‐selective electrodes.

In CV measurements, both ions and electrons are transferred, and if the system is electrochemically reversible – as will be shown later in Figure 2 – the mid‐peak potential corresponds to the total Gibbs energy of both processes.

### The One‐electron Transfer Reaction

In alkaline solution, when the proton insertion/deinsertion reaction (Scheme 2a) is at equilibrium state, the OCP from potentiometry and the reversible potential (*E*_rev_) from CV are described in the following sections.

#### Potentiometric Measurement (OCP)

**Scheme 3:**
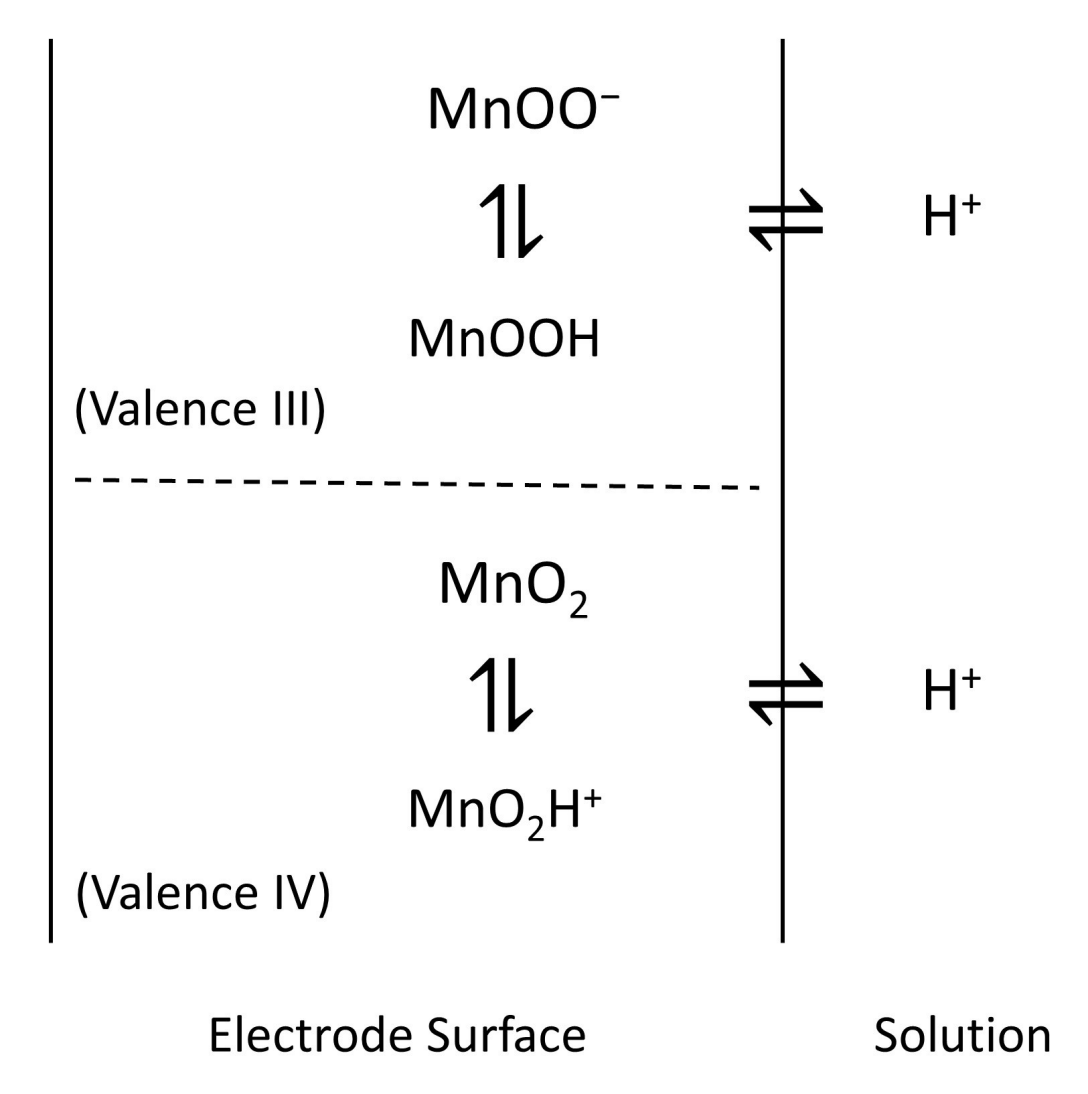
A model for proton transfer equilibrium of the MnO_2_ electrode at mid‐peak potential.

After applying the mid‐peak potential (*E*_mp_) of the MnO_2_/MnOOH species to the electrode for some time, both solid species must exist at equal activities. Hence, the OCP signal of the electrode can be depicted as Scheme 3 with the following equilibrium reactions (IT, ET, and {} symbols stand for ion transfer, electron transfer, and solid phase):

For the IV‐valent species:

(3) $$\left\{{MnO}_{2}\right\}+H^{+}\vec{\leftarrow }\left\{{MnOOH}^{+}\right\}$$

(4) $$E_{IT,\;\;IV}=E_{IT,\;\;IV\;}^{0}+\frac{RT}{F}ln\frac{a_{\left\{{MnO}_{2}\right\}}}{a_{\left\{{MnOOH}^{+}\right\}}}+\frac{RT}{F}lna_{H^{+}}$$

Similarly for the III‐valent species:

(5) $$\left\{{MnOO}^{-}\right\}+H^{+}\vec{\leftarrow }\left\{MnOOH\right\}$$

(6) $$E_{IT,\;\;III}=E_{IT,\;\;III\;}^{0}+\frac{RT}{F}ln\frac{a_{\left\{{MnOO}^{-}\right\}}}{a_{\left\{MnOOH\right\}}}+\frac{RT}{F}lna_{H^{+}}$$

Then, the OCP is the average of the two equilibrium potentials (Eq. 4 and 6:

(7) $$E_{OCP}=\frac{1}{2}\left[E_{IT,\;\;III}+E_{IT,\;\;IV}\right]$$

(8) $$E_{OCP}=\frac{E_{IT,\;\;IV\;}^{0}+E_{IT,\;\;III\;}^{0}}{2}+\frac{RT}{2F}ln\frac{{a_{\left\{{MnOO}^{-}\right\}}a}_{\left\{{MnO}_{2}\right\}}}{a_{\left\{MnOOH\right\}}a_{\left\{{MnOOH}^{+}\right\}}}+\frac{RT}{F}lna_{H^{+}}$$

Since at mid‐peak potential the activity of III‐ and IV‐valent species are equal, i. e., $a_{\left\{{MnO}_{2}\right\}}=a_{\left\{MnOOH\right\}}$ and $a_{\left\{{{Mn}^{III}OO}^{-}\right\}}=a_{\left\{{{Mn}^{IV}OOH}^{+}\right\}}$ :

(9) $$E_{OCP}=\frac{E_{IT,\;\;IV\;}^{0}+E_{IT,\;\;III\;}^{0}}{2}+\frac{RT}{F}lna_{H^{+}}$$

We assume a topotactic electrochemical reaction for MnO_2_, i. e., the crystal structure of MnO_2_ (hexagonal) and MnOOH (orthorombic) are similar, which may be verified by recording OCP vs. state of discharge (See Sec. 2.2, ESI). Thus, the first term in Eq. 9 is the average potential ($E_{IT}^{0}$ ) of two close potentials. Hence, Eq. 9 simplifies to:

(10) $$E_{OCP}=E_{IT}^{0}-0.059\left[V\right]pH\;\left(\mathrm{at}\;25\;{}^{\circ }C\right)\;$$

#### CV Measurement

The overall reaction in CV is as follows:

(11) $$\left\{{MnO}_{2}\right\}+e^{-}+H^{+}\vec{\leftarrow }\left\{MnOOH\right\}$$

Which can be dissected to ET and IT reactions:

(12) $$ET:\;\left\{{MnO}_{2}\right\}+e^{-}\vec{\leftarrow }\left\{{MnOO}^{-}\right\}$$

(13) $$E_{ET}=E_{ET}^{0}+\frac{RT}{F}ln\frac{a_{\left\{{MnO}_{2}\right\}}}{a_{\left\{{MnOO}^{-}\right\}}}$$

(14) $$IT:\;\left\{{MnOO}^{-}\right\}+H^{+}\vec{\leftarrow }\left\{MnOOH\right\}$$

(15) $$E_{IT}=E_{IT}^{0}+\frac{RT}{F}ln\frac{a_{\left\{{MnOO}^{-}\right\}}a_{H^{+}}}{a_{\left\{MnOOH\right\}}}$$

For the overall reaction:

$$E_{rev}=E_{ET}+E_{IT}$$

$$=E_{ET}^{0}+\frac{RT}{F}ln\frac{a_{\left\{{MnO}_{2}\right\}}}{a_{\left\{{MnOO}^{-}\right\}}}+E_{IT}^{0}+\frac{RT}{F}ln\frac{a_{\left\{{MnOO}^{-}\right\}}}{a_{\left\{MnOOH\right\}}}+\frac{RT}{F}lna_{H^{+}}$$

(16) $$=E_{ET}^{0}+E_{IT}^{0}+\frac{RT}{F}ln\frac{a_{\left\{{MnO}_{2}\right\}}}{a_{\left\{MnOOH\right\}}}+\frac{RT}{F}lna_{H^{+}}$$

At mid‐peak potential:

(17) $$E_{mp}=E_{ET}^{0}+E_{IT}^{0}+\frac{RT}{F}lna_{H^{+}}$$

Hence:

(18) $$E_{mp}=E_{ET}^{0}+E_{IT}^{0}-0.059\left[V\right]pH\;\left(\mathrm{at}\;25\;{}^{\circ }C\right)\;$$

In the above treatment, it is also possible to write first the IT and then the ET step, but the result is the same. Now by subtracting the equations obtained for the OCP and the mid‐peak potential (Eq. 10 and 18:

(19) $$E_{mp}-E_{OCP}=\left[E_{ET}^{0}+E_{IT}^{0}-0.059pH\right]-\left[E_{IT}^{0}-0.059pH\right]$$

(20) $$E_{mp}-E_{OCP}=E_{ET}^{0}$$

The $E_{mp}$ can be accessed from the CV for a reversible electrochemical reaction, and the $E_{IT}^{0}$ can be accessed by OCP measurements after preconditioning at mid‐peak potential of the electrode. Accordingly, the $E_{ET}^{0}$ term for the proton insertion/deinsertion reaction can be calculated from their subtraction. Electrochemically, this holds true as long as the same ions responsible for the OCP would cross the interface during the CV measurement to maintain charge neutrality in the solid phase. In order to be able to experimentally verify this situation and access the reversible potentials at standard condition, we make two approximations: 1) the activities are equal to concentrations, and 2) the diffusion coefficients are comparable (See Sec. 2.3, ESI).^[13]^

### The Two‐electron Transfer Reaction

In acidic solution, when the two‐electron transfer reaction is at equilibrium state (Eq. 2, and Scheme 2b), the overall reaction (i. e., ion plus electron transfer) can be described by the following Nernst equation:

(21) $$E_{rev}=E_{rev}^{0}+\frac{RT}{2F}ln\frac{a_{H^{+}}^{4}}{a_{{Mn}^{2+}}}$$

(22) $$E_{rev}=E_{rev}^{0}-29.6\left[mV\right]loga_{{Mn}^{2+}}-118.2\left[mV\right]pH\;\left(\mathrm{at}\;25\;{}^{\circ }C\right)\;$$

Vetter et al. and Covington et al have systematically studied this reaction by OCP measurements. Vetter has assumed an Mn^2+^ transfer across the interface for this equilibrium (interfacial transfer of either Mn^2+^, H^+^ or Mn^2+^, O^2−^ couples),^[12]^ and similarly Covington has assumed an ion‐exchange capacity for H^+^ and Mn^2+^ for the OCP measurements.^[14]^ Nonetheless, here we assume that the reaction in the OCP measurement has a redox‐type nature, i. e., the dissolution of MnO_2_ in reaction with H^+^ ions. However, in the presence of an Mn^2+^ concentration added in the solution, the spontaneous dissolution process is inhibited (i. e., reduced forward rate and increased backward rate) so that it is possible to measure practically‐stable OCPs, and assume an equilibrium state under which Eq. 22 may be verified.

In the CV measurement, this reaction can be pictured as the reductive dissolution of MnO_2_ to Mn^2+^ near the electrode surface and in the reverse scan as the oxidative re‐deposition of Mn^2+^ to MnO_2_. Therefore, both OCP and *E*_mp_ are described by the same relationship (Eq. 22), and the ion transfer and electron transfer potentials (or Gibbs energies) cannot be separated by the method described above.

### OCP Studies

**Figure 1:**
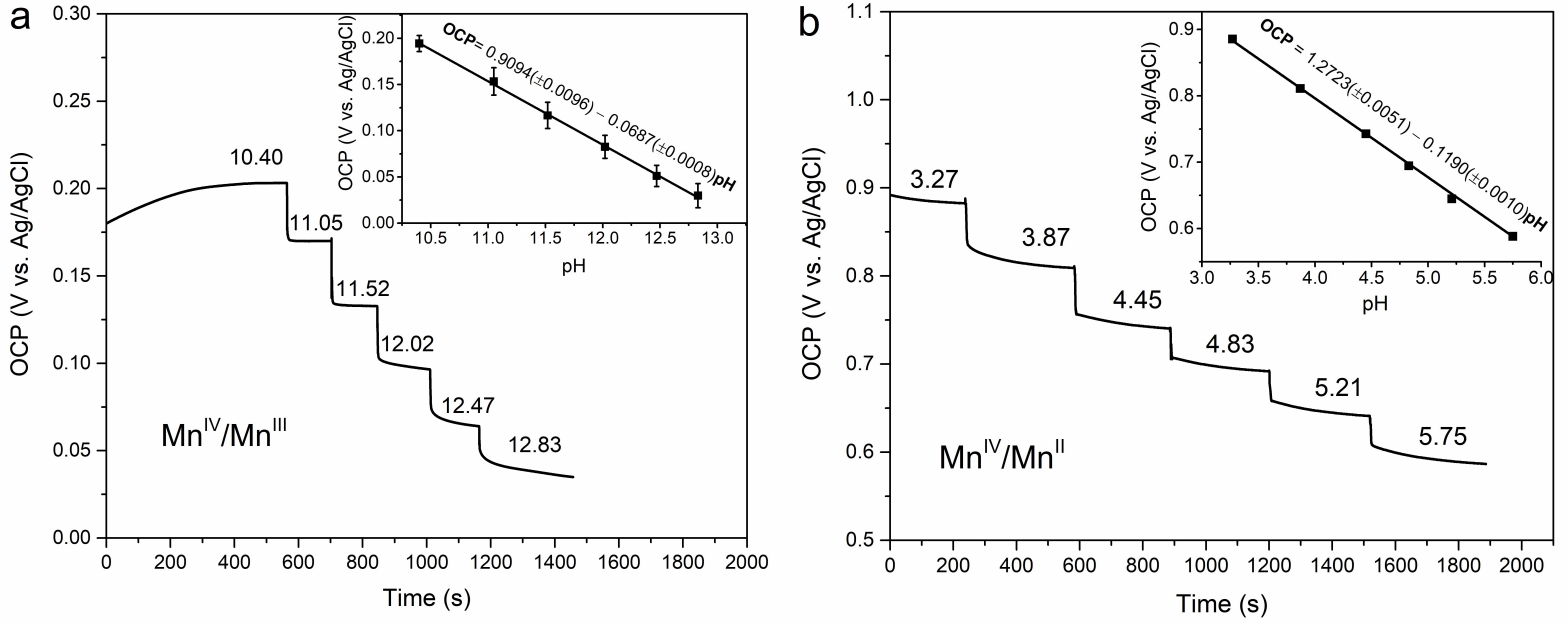
Effect of solution pH on the OCP of MnO_2_ after application of the respective formal potentials in alkaline and acidic solutions in a) 0.01 M K_2_SO_4_+KOH solution and b) 0.02 M acetate buffer solution containing 0.01 mM Mn^2+^ (each point in the inset curves is the average of 3 to 5 measurements with different electrodes)

Before OCP measurements, the formal potential for the redox reaction (determined from CV measurements) was applied to the electrodes for 60 s to condition the electrodes so that both MnO_2_ and MnOOH exist at equal activities. Figure 1a and b depict the open circuit potentiometry of the MnO_2_ electrode at different solution pH in alkaline and acidic solutions, respectively. The slopes of 68.7 and 122.7 mV dec^−1^ for the OCP vs. pH plots (inset curves) are in good agreement with the Nernst equations described above for the proton insertion/deinsertion and two‐electron transfer reactions. The OCP response of MnO_2_ in acidic solution to different Mn^2+^ concentrations may also be recorded (Sec. 2.4, ESI) which produces a slope of ∼30 mV dec^−1^ in agreement with Eq. 22. Comparing the OCP‐pH equation in Figure 1b with Eq. 22 gives a standard potential $E^{0}$ of 1.322 V for this reaction of ϵ‐MnO_2_. Covington et al^[14]^ by using a similar potentiometric method and accounting for the activity coefficients have reported the standard potential of the β‐MnO_2_ to be 1.238 to 1.242 V which is 82 mV lower than the value measured here for ϵ‐MnO_2_. Using the Covington value of $E^{0}$ for β‐MnO_2_ and the $E^{0}$ value measured here for ϵ‐MnO_2_, the standard Gibbs energies of formation are calculated to be −462.7 kJ mol^−1^ and −427.3 kJ mol^−1^, respectively. Since β‐MnO_2_ is the thermodynamically most stable phase of MnO_2_, these formation energies indicate that the ϵ‐MnO_2_ is less stable by 35.4 kJ mol^−1^, i. e.,

(23) $$\epsilon -MnO_{2}\to \beta -MnO_{2}\;\Delta G_{transform.}^{0}=-35.4\;\;kJ\;{mol}^{-1}$$

This phenomenon could be attributed to the abundant Mn^4+^ vacancies of ϵ‐MnO_2_ and its different coordination environment.

### Voltammetric Studies

**Figure 2:**
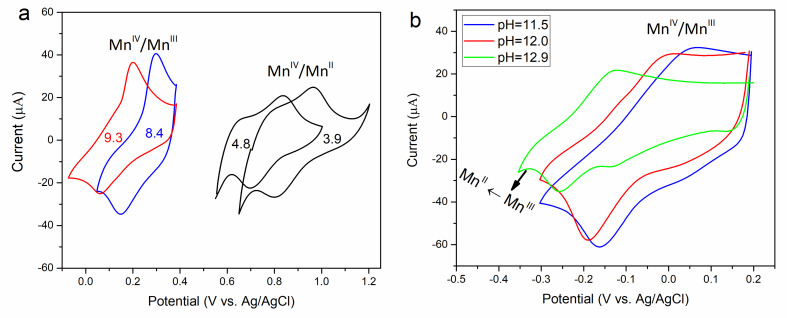
The effect of solution pH on the CV curves of ϵ‐MnO_2_. a) CV curves in 0.02 M acetate buffer solution containing 0.01 mM Mn^2+^ at 2.5 mV s^−1^ (black) and in 0.1 M ammonium buffer solution at 2 mV s^−1^ (blue and red) b) CV curves in KOH+0.01 M K_2_SO_4_ at 2 mV s^−1^.

The effect of solution pH on the MnO_2_ electrode was also studied by cyclic voltammetry (CV). Figure 2a shows the CV curves of MnO_2_ in acidic solution containing 0.01 mM Mn^2+^ with pH values of 3.9 and 4.8 (black curves) and alkaline solution with pH values of 8.4 (blue curve) and 9.3 (red curve). In acidic solution, the curves show an *E*_mp_ vs. pH slope of ∼120 mV dec^−1^ with a peak‐to‐peak separation of ∼146 mV which is somewhat above that expected for a reversible two‐charge transfer reaction. The reduction at the lower limit of the CV (black curves) is due to irreversible bulk reduction of MnO_2_ to Mn^2+^ (Figure S4a, ESI,). In alkaline solution, the CV curves show an *E*_mp_ vs. pH slope of ∼60 mV dec^−1^ with a peak‐to‐peak separation of ∼125 mV, implying a (quasi) reversible one‐electron transfer reaction. Furthermore, the active mass of MnO_2_ calculated by integrating the Red/Ox peaks for each reaction is compared with the total mass of deposited MnO_2_ (Sec. 2.6, ESI). They indicate that only 3.5 wt.% of the total MnO_2_ participates in the two‐electron transfer reaction in acidic solution, but 31.0 wt.% participates in the proton insertion/deinsertion reaction in alkaline solution, in agreement with near‐surface and bulk processes, respectively.

Figure 2b exhibits the CV curves of the MnO_2_ electrode in 0.01 M K_2_SO_4_+KOH solution at higher pH values. The CV mid‐peak potentials still show a slope of ∼60 mV dec^−1^ up to the pH of ∼12.4. At pH=12.9 (green curve), part of the MnOOH is reduced to Mn(OH)_2_ at around −0.3 V, with a more negative *E*_mp_. A reaction mechanism for the reduction of MnO_2_ to Mn(OH)_2_ under strong alkaline solutions has been proposed by Yeager et al..^[15]^

**Figure 3:**
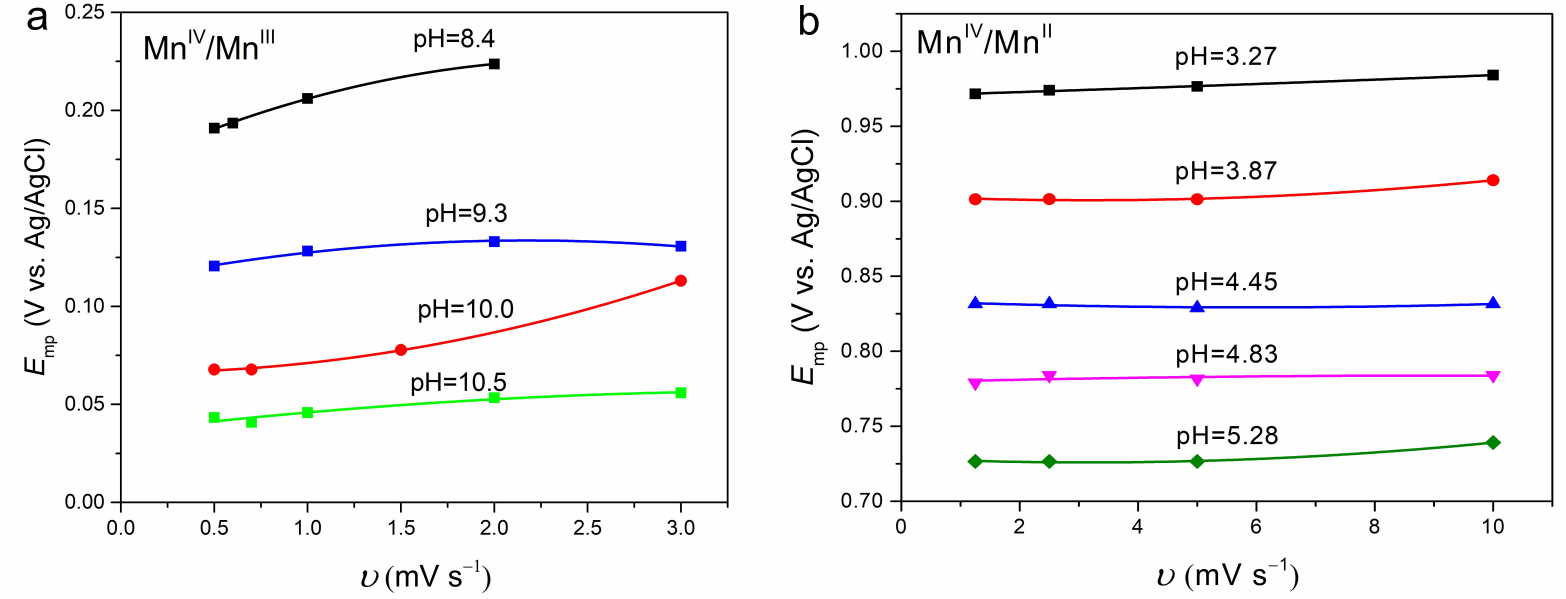
Effect of scan rate on the mid‐peak potentials (*E*_mp_) of ϵ‐MnO_2_ in 0.1 M ammonium buffer (a) and 0.02 M acetate buffer+0.01 mM Mn^2+^ (b) with different solution pH.

As noted, the CV curves of the proton insertion/deinsertion reaction and the two‐electron transfer reaction of ϵ‐MnO_2_ deviate from those of ideal reversible reactions. Therefore, to remove the possible kinetic effects on the mid‐peak potentials (*E*_mp_), they are extrapolated to the scan rate of zero. Figure 3a and b exhibit the *E*_mp_ values obtained at different scan rates for each pH in alkaline and acidic solution, respectively. The *E*_mp_‐ν curves for both reactions are fitted to quadratic equations, and the extrapolated *E*_mp_ was used as the *E*_mp_ of the reversible reaction at the corresponding pH for the next analysis.

**Figure 4:**
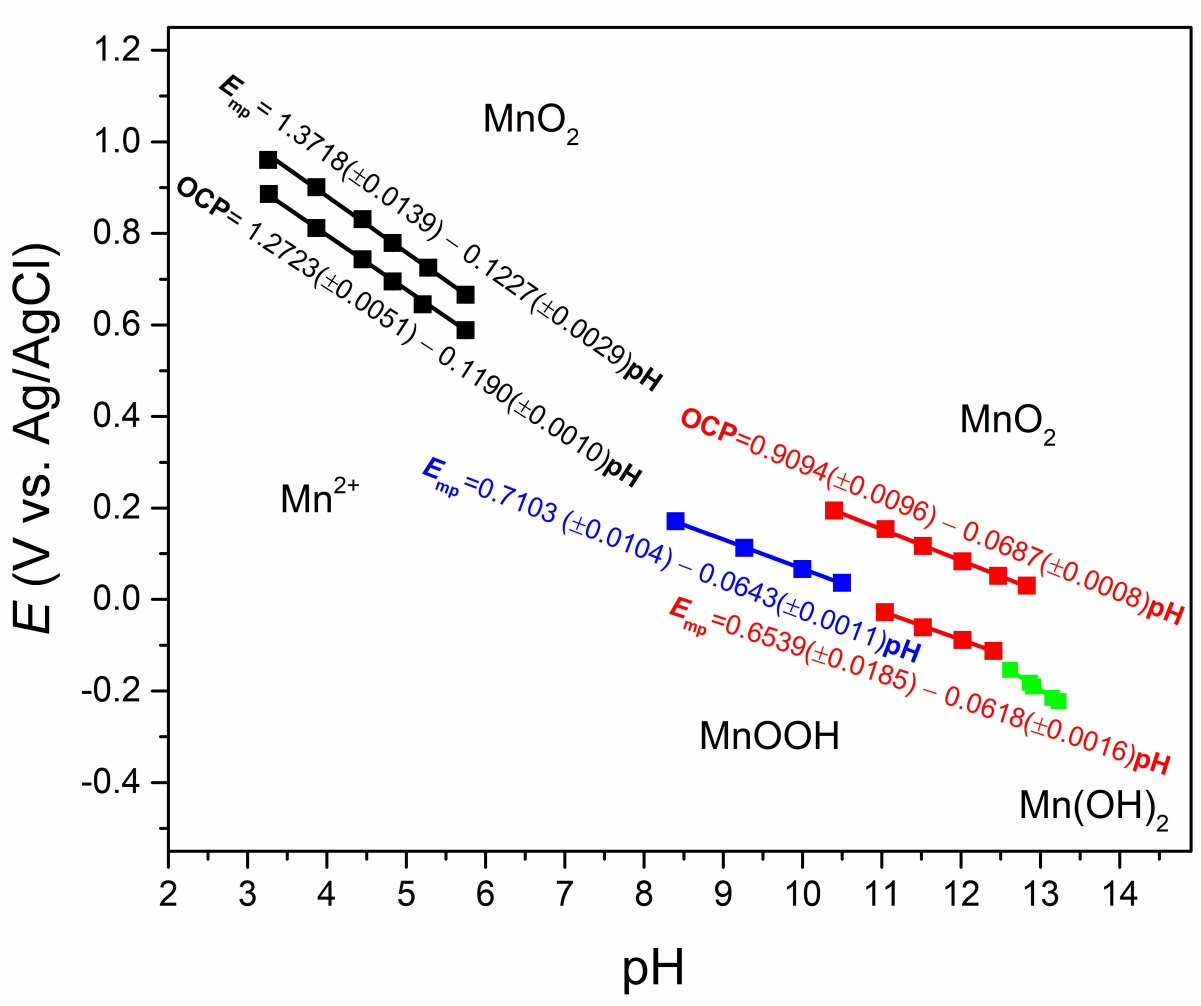
The *E*‐pH diagram of ϵ‐MnO_2_ based on the *E*_mp_ and OCP measurements. (black: acetate buffer, blue: ammonium buffer, red: KOH solution, green: mixed behaviour in KOH).

Figure 4 displays the as‐obtained *E*_mp_–pH curves along with previously‐obtained OCP–pH curves in one plot in resemblance to the MnO_2_ phase‐diagram. The slope of *E*_mp_–pH curve for the proton insertion/deinsertion reaction is 64.3 mV dec^−1^ in ammonium buffer solution (blue curve) and 61.8 mV dec^−1^ in KOH solution (red curve). Here the former curve is selected over the other, because it is obtained from the results of the extrapolations while the latter is constructed from averaging the *E*_mp_ values of different electrodes without extrapolation (i. e., by repeating the electrode preparation and the measurement five times).

Now that the $E_{rev}^{0}$ , $E_{IT}^{0}$ , and $E_{ET}^{0}$ values became available from the intercepts of the *E*–pH plot for the proton insertion/deinsertion reaction, the corresponding standard Gibbs energies can also be calculated from ${\Delta G}^{0}=-nFE^{0}.$ ^[4]^ The standard Gibbs energy of electron transfer is already evident from the gap between parallel curves of OCP–pH and *E*_mp_–pH (Figure 4) which are not affected by proton concentration as expected.

Table 1 compares the individual and overall standard Gibbs energies of ion and electron transfer for ϵ‐MnO_2_. The overall Gibbs energy of the two‐electron transfer reaction is 3.1 times more negative than that of the proton insertion/deinsertion reaction, because during the reduction of MnO_2_ to Mn^2+^ the ingress of H^+^ ions and the egress of O^2−^ ions from the oxide form four covalent O−H bonds, resulting in the egress of two H_2_O and hydration of one Mn^2+^ ion. Thus its very negative standard Gibbs energy has an enthalpic nature, i. e., it is an enthalpy‐driven reaction as it can be calculated (See Sec. 2.7, ESI).

**Table 1:** Gibbs energies in kJ mol^−1^ calculated for ion transfer, electron transfer, and the overall process for the proton insertion/deinsertion reaction in ϵ‐MnO_2_ in aqueous solution at 25 °C. (the overall Gibbs energy of the two‐electron transfer reaction is also indicated for comparison).

	H+ (de)insertion reaction	Two‐electron transfer reaction
${\Delta G}_{IT}^{0}$	−106.8 (±0.9)	–
${\Delta G}_{ET}^{0}$	+19.2 (±1.3)	–
${\Delta G}_{overall}^{0}$	−87.5 (±1.0)	−274.2 (±2.7)

On the other hand, during the proton insertion reaction, the ϵ‐MnO_2_ (hexagonal) is transformed to α‐MnOOH with orthorhomobic crystal structure.^[16]^ The ${\Delta G}_{ET}^{0}$ of this reaction (+19.2 kJ mol^−1^) is unfavorable because the electron insertion converts the Mn^4+^ to Mn^3+^ ions in the solid phase from the ionic radii of 0.530 Å to 0.645 Å,^[17]^ respectively, creating a lattice cell expansion. This is understandable with regard to the fact that the proton insertion alone converts O^2−^ to OH^−^ ions which have identical radii, and hence, cannot cause a lattice expansion. Furthermore, the large ${\Delta G}_{IT}^{0}$ for the H^+^ insertion/deinsertion (−106.8 kJ mol^−1^) indicates a high affinity of protons for the oxygen groups located in the tunnels of ϵ‐MnO_2_ and its dominant role in determining the overall Gibbs energy.

**Scheme 4:**
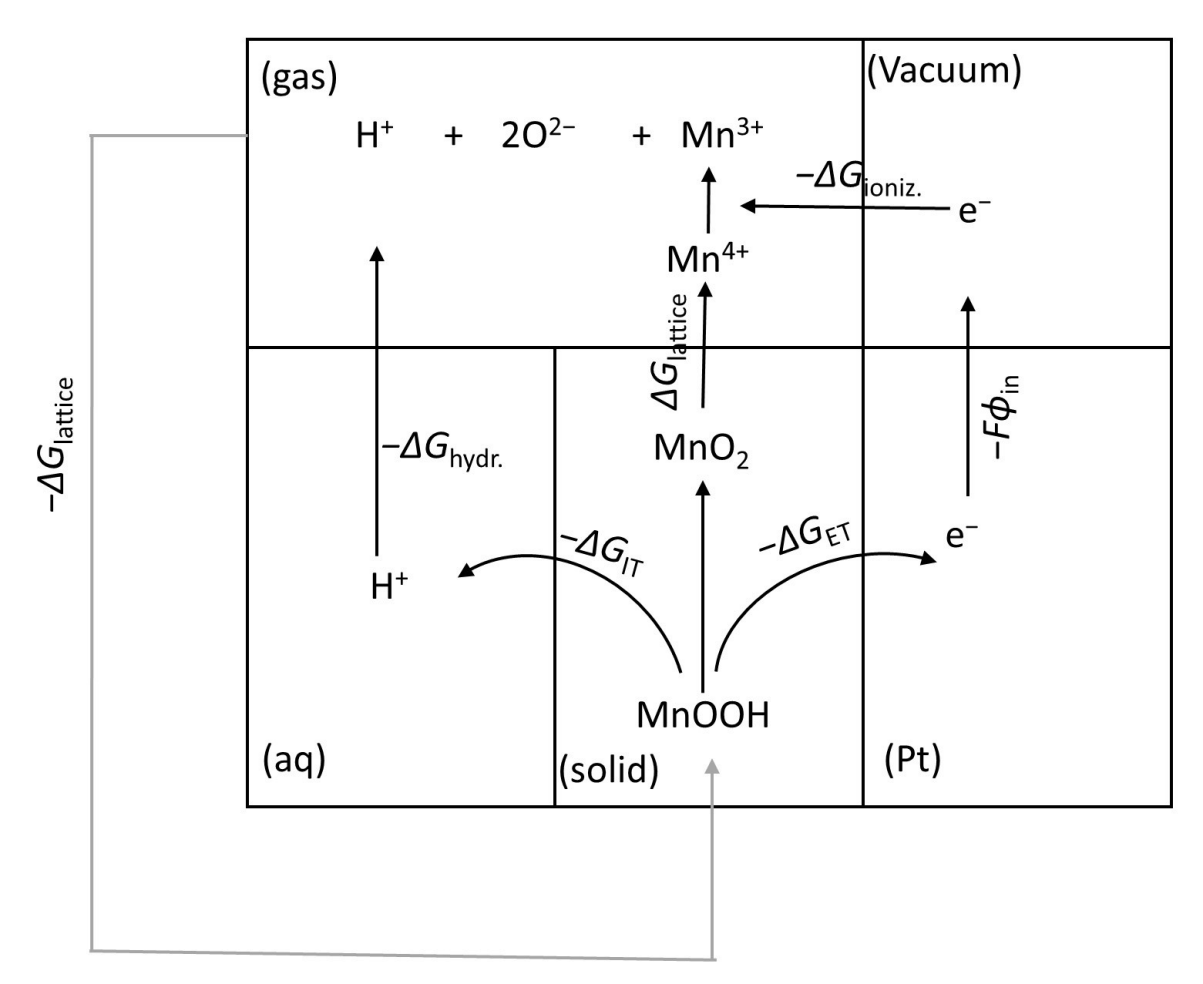
A thermochemical cycle for the proton insertion/deinsertion reaction in MnO_2_ in conjuction with aqueous solution.

Finally, Scheme 4 displays a thermochemical cycle for the proton insertion/deinsertion model of ϵ‐MnO_2_. The dark arrows show the electrochemical route for ionizing the MnOOH. Thus the lattice energy of MnOOH (gray arrow) should close the overall cycle:

(24) $${\Delta G}_{hydr.\left(\;H^{+}\right)}^{0}+{\Delta G}_{IT}^{0}+{\Delta G}_{ET}^{0}+{\Delta G}_{lat.\left({MnO}_{2}\right)}^{0}+{\Delta G}_{ioniz.\left({Mn}^{3+}\to {Mn}^{4+}\right)}^{0}+W+{\Delta G}_{lat.\left(MnOOH\right)}^{0}=0$$

The ionization potential of Mn^4+^ (${\Delta G}_{ioniz.\left({Mn}^{3+}\to {Mn}^{4+}\right)}^{0}$ ) is 4940 kJ mol^−1^.^[19]^ By neglecting the entropy terms for ionization (i. e., Δ*G* ∼ Δ*H*), the crystal lattice energies of MnO_2_ and MnOOH can be calculated through Born‐Haber cycles from the ${\Delta G}_{overall}^{0}$ values determined here (Sec. 2.8 and 2.9, ESI). Furthermore, the work function (*W*) of polycrystalline Pt is equal to 5.64 eV.^[20]^ Hence:

$${\Delta G}_{hydr.\left(\;H^{+}\right)}^{0}+106.8-19.2+10048.3-4940+544.2-6753.8=0$$

From this summation, the standard Gibbs energy of hydration of proton ($-{\Delta G}_{hydr.\left(\;H^{+}\right)}^{0}$ ) is calculated to be −1013.7 kJ mol^−1^ which is in good agreement with the value −1056 kJ mol^−1^ available from thermodynamic tables.^[18]^

## Conclusion

To improve the performance of current ion‐insertion batteries, a fundamental understanding of the undergoing ion and electron transfer processes and their associated energetics across the electrode interfaces is essential. Here, two fundamentally‐different electrochemical reactions, i. e., the proton insertion/deinsertion reaction and the superficial two‐electron transfer reaction, for ϵ‐MnO_2_ in alkaline and acidic solutions were identified and verified. For the former reaction, the individual Gibbs energies of electron and proton transfer were determined for the first time based on the reversible potentials in cyclic voltammetry and open‐circuit potentiometry. These data indicate that the electron insertion is an unfavorable step but it is overcompensated by the favorable proton insertion into tunnel‐type ϵ‐MnO_2_. In addition, the determined individual Gibbs energies of proton and electron transfer across the interfaces of the ϵ‐MnO_2_ electrode agreed with the hydration Gibbs energy of proton in a thermochemical cycle.

Finally, the determination of individual standard Gibbs energies for electron and proton transfer may also be used to reveal the effect of the type of inserting ion (H^+^, Li^+^, Na^+^, …) and the solvent on the electrode performance, which could be a topic for a future study.

## Conflict of interest

The authors declare no conflict of interest.

1

## Supporting information

As a service to our authors and readers, this journal provides supporting information supplied by the authors. Such materials are peer reviewed and may be re‐organized for online delivery, but are not copy‐edited or typeset. Technical support issues arising from supporting information (other than missing files) should be addressed to the authors.

Supporting Information

## Data Availability

The data that support the findings of this study are available in the supplementary material of this article.
