# Supplementary material for: Determining the Gibbs Energy Contributions of Ion and Electron Transfer for Proton Insertion in ϵ‐MnO2
Source: Chemphyschem. 2022 Oct 17;23(24):e202200364. doi: 10.1002/cphc.202200364 (PMC10092740; doi:10.1002/cphc.202200364)
Supplement: Supplementary file 1 — Supporting Information [file CPHC-23-0-s001.pdf]

ChemPhysChem

Supporting Information

Determining the Gibbs Energy Contributions of Ion and Electron Transfer for Proton Insertion in ε-MnO_2

Keyvan Malaie,* Fritz Scholz, Uwe Schröder,* Harm Wulff, and Heike Kahlert

1 Experimental

1.1 Material and methods

All the chemicals were from Sigma-Aldrich Company and used as received.

1.2 Instrumentation for XRD and electrochemical measurements

The crystalline structures of the powders were identified by X-ray diffraction (XRD) on a Bruker D8 Advance X-ray diffractometer using Cu Kα X-ray source radiation (λ= 1.54 05 Å).

The electrochemical measurements were carried out in a three-electrode configuration in aqueous solution using cyclic voltammetry (CV) and open-circuit chronopotentiometry on an Autolab instrument. Pt rods (~0.38 mm diameter, and 0.29-36 cm^{-2}) were used as current collector for the electrochemical deposition of the samples. A Pt coil and a Ag/AgCl (saturated KCl) electrode were used as counter and reference electrodes in aqueous solution, respectively. A salt bridge consisted of Agar and saturated KNO_3 is used to avoid chloride leakage and also to reduce the junction potential. The potential of the reference electrode is 197.0 V vs. standard hydrogen electrode (SHE), the faraday constant is 96485.34 C mol^{-1}, and 1 eV is 96.483 kJ mol^{-1}. Where the reference electrode is not indicated for the potential, the SHE is meant.

1.3 CV measurements

In acidic solution, the CV measurements were carried out in acetate buffer solution (ABS, 0.02 M, pH = 3.27-5.75) containing 0.01 mM Mn^{2+}; and in alkaline solution they were carried out in KOH + 0.05 M KCl its pH is regulated by a 10 M KOH solution (KOH concentration in the solution: 0.002−0.1 M). The first cycle of the CV was used to obtain the E−pH curves and each point averaged from 3 to 5 measurements from different electrodes. The E_{mp} values of the CV were recorded at different scan rates from 0.5 to 10 mV s^{-1} for each solution pH and extrapolated to a scan rate of zero. The pH of the solution was measured by the laboratory glass pH meter (model Knick, 761 Calimatic) and calibrated with standard buffer solutions (pH = 4.00 and 7.00 $\pm$0.02) from Carlroth Co. before each set of measurements.

1.4 OCP measurements

The OCP curves were recorded under the same condition as that of the CV measurements. Before OCP measurements, the formal potential for the redox reaction (determined from CV measurements) was applied to the electrodes for 60 s. Next, the OCP values were recorded after they reached a constant value that does not change above 1 mV over the following 1-2 minutes (or below 10 mV per hour). In the measurement of the two-electron transfer reaction of MnO_2 in acidic solution, Mn^{2+} concentration is fixed to 0.01 mM in all experiments, otherwise the OCP could not stabilize due to the ongoing MnO_2 dissolution. On the other hand, the OCP measurements in alkaline solution (either KOH/0.01 M K_2SO_4 or 0.1 M ammonium sulfate buffer solution) were performed in stringent solutions with no Mn^{2+} ions in the solution.

1.5 Material synthesis and electrode preparation

For the electrodeposition of the samples, first the Pt rods were cleansed carefully in aqua regia for 5 to 10 minutes. Then, they are washed with deionized water (DI). The electrodeposition was conducted by potentiostatic mode at room temperature (25.0 °C) on the Pt rods from aqueous solutions de-aerated by N_2 for 10 minutes. The ε-MnO_2 sample was deposited anodically from a solution of 40 mM $MnSO_4$ + 50 mM H_2SO_4 at a potential of 1.25 V (vs. Ag/AgCl) for 20 s. After deposition, the electrodes were cleansed with DI water and left to dry at room temperature for 30 minutes.

2 Results and Discussion

2.1 XRD analysis of the ε-MnO_2 sample

Fig. S1 shows the XRD pattern of the ε-MnO_2 powder sample that is scratched from the Pt substrate. The fundamental reflections of ε-MnO_2, i.e., (100), (002), (101), (102), and (110), are all observed for our MnO_2 sample, with (100) and (110) being the most intensive[1]. In addition, the XRD pattern does not show any extra reflections

from other MnO_2[2] phases. The unassigned broad peak at ~22 ° 2θ (~0.42 nm) is observed in most commercial electrolytic manganese dioxides (EMD) that contain roughly 44–50% Ramsdellite and 44–51% ε-MnO_2 phases. According to Kim et al, who have carried out a detailed analysis of ε-MnO_2 samples, this anomalous peak appears in materials containing appreciable concentrations of ε-MnO_2 due to the strong (110) superlattice reflection[3]. The calculated lattice parameters for ε-MnO_2 are a = 0.2826 nm and c = 0.4453 nm, almost equal to ICSD data (FIZ Karlsruhe 2021).

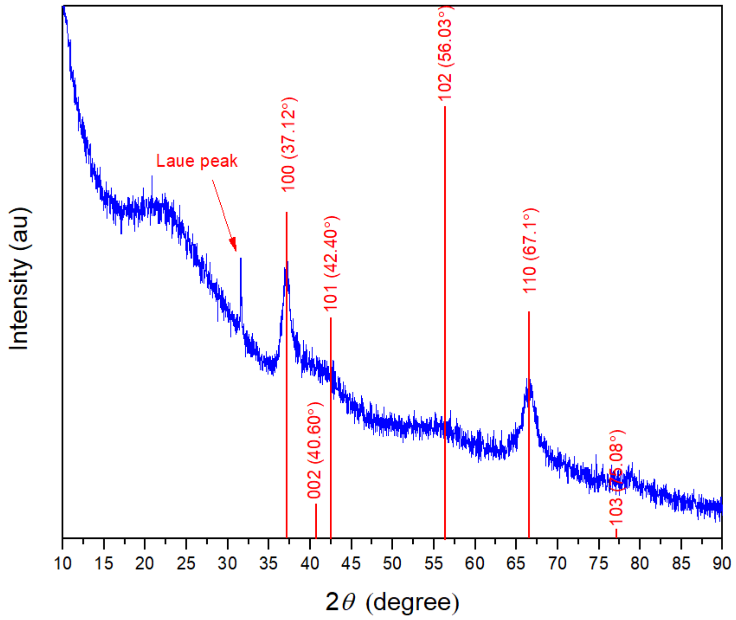

Fig. S1 Comparison of the XRD pattern of ε-MnO_2 fine powder prepared by electrochemical deposition (blue) and the calculated XRD pattern of ε-MnO_2 (red). The space group is P63/mmc (194)

2.2 OCP vs. state of discharge for the proton insertion/deinsertion reaction

In order to show how the OCP of the MnO_2 electrode differs from the crystal phase of MnO_2 (hexagonal) to the crystal phase of MnOOH (orthorhombic), the MnO_2 electrode is reduced incrementally by applying the potentials 0.2, 0.1, 0, −0.1, and −0.2 V for 60 s in KOH with pH = 12.0 as displayed in Fig. S2. It indicates that the OCP changes only by ~20 mV between the two crystal phases.

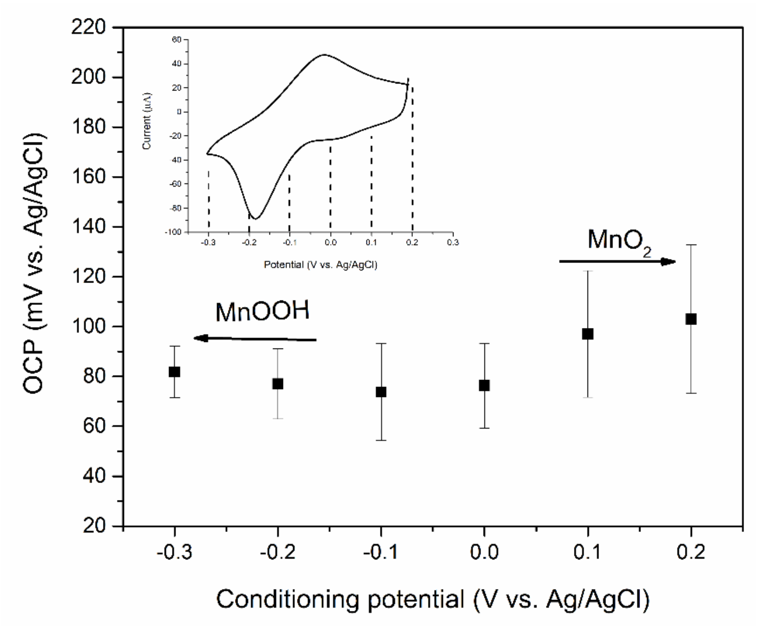

Fig. S2 OCP measurements after conditioning the MnO_2 electrode at different potentials for 60 s in KOH solution with pH=12.0. inset figure is the CV of the same electrode at 2 mV s^{-1} under the same condition that is used for choosing the conditionig potentials. (each point is an average of three measurements with three electrodes)

2.3 Estimation of formal potential from mid-peak potential

In contrast to OCP (E_{OCP}), the CV mid-peak potentials (E_{mp}) contain also the diffusion coefficient terms for the soluble Red and Ox species. However, here the redox species are solid and only the inserting ions have a diffusion coefficient. The proton insertion is associated with reduction and the proton extraction is associated with oxidation. Therefore, if both ion insertion and extraction diffusion coefficients are comparable, their effect on the CV mid-peak potentials is negligible. This assumption makes sense for the proton insertion/extraction of MnO_2 especially with regards to the rather symmetric CV peaks vs. E_{mp} point.

2.4 OCP response of MnO_2 to Mn^{2+}

The OCP response of the MnO_2 electrode to Mn^{2+} concentration is studied with two slightly-different electrodes, i.e., the as-prepared MnO_2 (fresh) and the same electrode after storing it in 0.01 M acetic acid (pH=3.4) for two days (aged) in order to check if the ongoing spontaneous dissolution of MnO_2 affects its surface structure. As exhibited by the OCP vs. time curves in Fig. S3a, the OCP of the aged electrode stabilizes at a more positive potential (red curve). However, both electrodes exhibit the same OCP response to different Mn^{2+} concentration with high stability vs. time, implying that the two electrodes have similar surface structures and that Mn^{2+} ions inhibit the spontaneous dissolution of the MnO_2.

The slope of OCP vs. Mn^{2+} concentration at a rather fixed pH is determined to be 42 ± 2.5 mV dec^{-1} (Fig. S3b). During this measurement the pH increases by ~0.26; thus if we account for this pH change, the corrected slope is 27.1 mV dec^{-1}. Therefore, this slope is in good agreement with the proposed Nernst equation at 25 °C (Eq. 22, main text). By assuming this equation, the E^0_{rev} is approximated to be 1.302 V for the MnO_2 in its fully oxidized state.

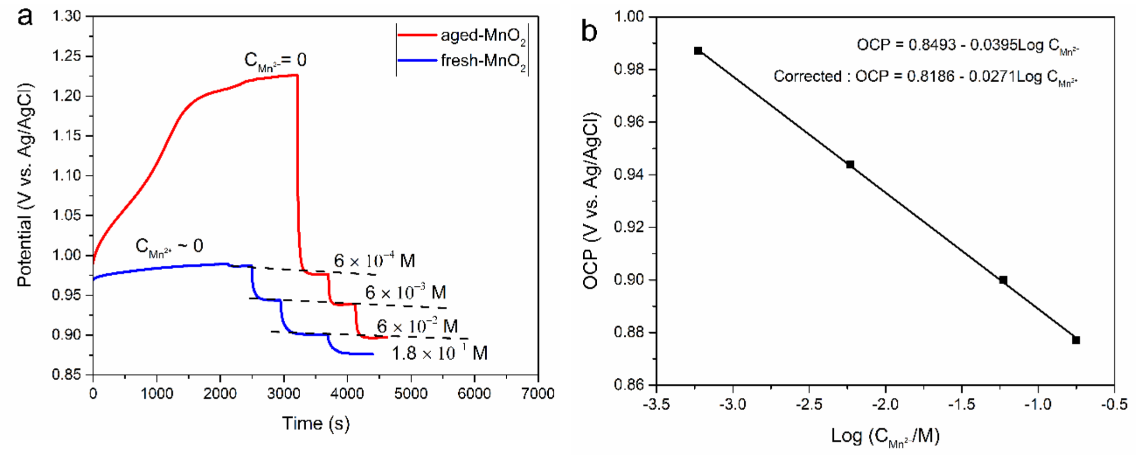

Fig. S3 a) Open−circuit chronopotentiometry of fresh (blue) and aged (red) MnO_2 in Robinson buffer solution (RBS, pH=2.42 ± 0.13) with different Mn^{2+} concentrations and b) OCP vs. Mn^{2+} concentration for MnO_2 electrode without aging. (the linear equation was corrected to account for potential change due to the pH change.

2.5 Voltammetric study of the two-electron transfer reaction in acidic solution

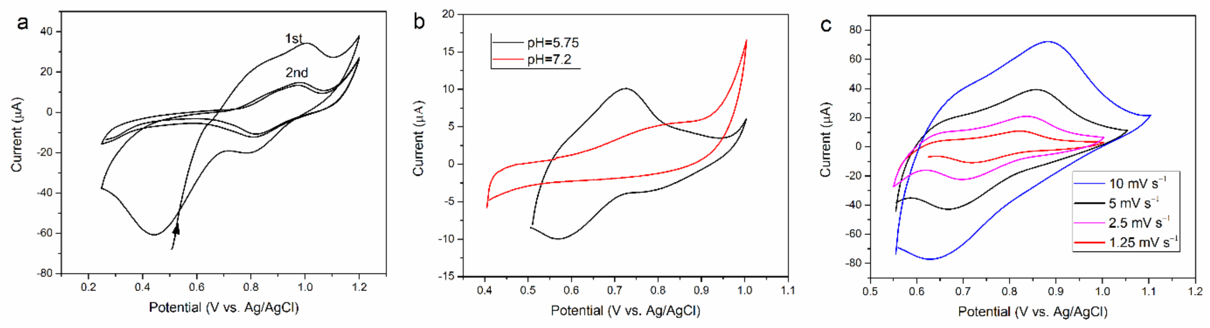

Fig. S4 CV curves of MnO_2 in 0.02 M acetate solution containing 0.01 mM Mn^{2+} a) the first three cycles at pH = 3.9 and 2 mV/s in a wide potential window b) CV curves at 2.5 mV/s for pH 5.75 and 7.2 showing that the two-electron transfer reaction does not extend to the neutral solution. c) CV curves at pH=4.8 at different scan rates.

2.6 Percent active mass of total MnO_2

In order to calculate the mass of deposited MnO_2 (anhydrate), the chronoamperometry curve recorded during the potentiostatic deposition is integrated and the same two-electron transfer reaction is assumed. The charge and mass calculated in this way may contain a slight contribution from Pt oxidation and oxygen evolution side reactions. For the calculation of the active mass of MnO_2, the Ox and Red peaks in the CV curves are integrated and averaged. For the two-electron transfer and proton insertion/deinsertion reactions, the CV curves recorded at pH = 3.4 and 9.3 are used, respectively (Table S1).

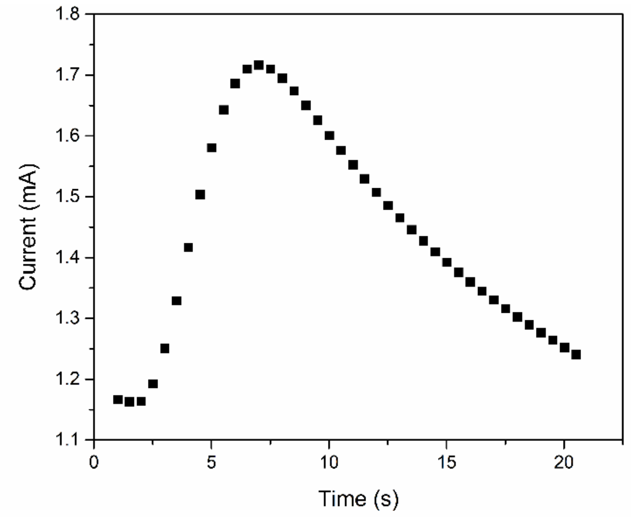

Fig. S5 Chronoamperometric curve recorded during the potentiostatic deposition of MnO_2 on the Pt wire at 1.25 V.

Table S 1 Mass of anhydrate MnO_2 calculated by integration from chronoamperometry and the redox peaks in CV

	Chrononamperometry	Two-electron transfer reaction		Proton insertion/deinsertion reaction	
		Ox peak	Red peak	Ox peak	Red peak
Mass (μg cm^{-2})	35.8	1.7	0.8	13.7	8.5

2.7 Enthalpic and entropic contributions in the two-electron transfer reaction of MnO_2

When SHE is the anode, the overall two-electron transfer reaction is as follows:

$$\mathrm{MnO_2(s) + H_2(g) + 2H^+(aq) \rightarrow Mn^{2+}(aq) + 2H_2O\ (l)} \tag{1}$$

We assume a β-MnO_2 phase because the standard entropy of this phase is available. The standard Gibbs free energy of formation for β-MnO_2, H_2O, and Mn^{2+} are −465.2 [4], 237.2 [4], and 228.1 kJ mol^{-1} [4,5]; thus the standard Gibbs free energy of the reaction is:

$$\Delta G^0_{\mathrm{rxn}} = \sum \Delta G^0_{\mathrm{f,prod}} - \sum \Delta G^0_{\mathrm{f,react}} = [2 \times (-237.2) + (-228.1)] - [-465.2 + 2 \times (0) + (0)]$$
$$= -237.3\ \mathrm{kJ\ mol^{-1}} \tag{2}$$

And the standard entropy of β-MnO_2, H_2, H_2O, and Mn^{2+} are 53.05 [5], 130.6 [4], 69.9 [4], and −73.6 J mol K^{-1} [4] [1]; thus the standard entropy change of the reaction is:

$$\Delta S^0_{\mathrm{rxn}} = \sum \Delta S^0_{\mathrm{prod}} - \sum \Delta S^0_{\mathrm{react}} = [2 \times 69.9 + (-73.6)] - [53.05 + 130.6 + 2 \times (0)]$$
$$= -0.1174\ \mathrm{kJ\ mol\ K^{-1}} \tag{3}$$

Thus, the standard enthalpy change of the reaction is:

$$\Delta H^0_{\mathrm{rxn}} = \Delta G^0_{\mathrm{rxn}} + T\Delta S^0_{\mathrm{rxn}} = -237.3 + [298 \times (-0.1174)] = -272.3\ \mathrm{kJ\ mol^{-1}} \tag{4}$$

[1] i.e., the conventional standard molar entropy of hydration for Mn^{2+}

2.8 Calculation of the formation Gibbs energy of ε−MnO2 and its lattice energy

The standard Gibbs free energy of formation for ε−MnO2 ($\Delta G^0_{\mathrm{f,\varepsilon-MnO_2}}$) can easily be calculated by using the standard free energy of the two-electron transfer reaction that is measured in this work (main text, Table 1):

$$\varepsilon\text{-}\mathrm{MnO_2(s) + H_2(g) + 2H^+(aq) \rightarrow Mn^{2+}(aq) + 2H_2O\ (l)} \qquad \Delta G^0_{\mathrm{rxn}} = -274.2\ \mathrm{kJ\ mol^{-1}} \quad (5)$$

$$\Delta G^0_{\mathrm{rxn}} = \sum \Delta G^0_{\mathrm{f,prod}} - \sum \Delta G^0_{\mathrm{f,react}} = [2 \times (-237.2) + (-228.1)] - \left[\Delta G^0_{\mathrm{f,\varepsilon-MnO_2}} + 0 + 0\right]$$
$$= -274.2\ \mathrm{kJ\ mol^{-1}}$$

$$\Delta G^0_{\mathrm{f,\varepsilon-MnO_2}} = -427.3\ \mathrm{kJ\ mol^{-1}} \quad (6)$$

This standard Gibbs energy of formation can be used in a Born-Haber cycle to calculate the lattice energy (E_{lattice}) of ε−MnO2. (sub. and diss. stand for sublimation and dissociation):

1) $\mathrm{Mn(s) \rightarrow Mn(g)}$ $\quad \Delta G_{\mathrm{sub.}} = 238\ \mathrm{kJ\ mol^{-1}}$

2) $\mathrm{Mn(g) \rightarrow Mn^{4+}(g) + 4e^-}$ $\quad \Sigma I_{\mathrm{p}} = 10414\ \mathrm{kJ\ mol^{-1}}$

3) $\mathrm{O_2(g) \rightarrow 2O\ (g)}$ $\quad \Delta G_{\mathrm{diss.}} = 249\ \mathrm{kJ\ mol^{-1}}$

4) $\mathrm{2O\ (g) + 4e^- \rightarrow 2O^{2-}(g)}$ $\quad \mathrm{EA} = -640\ \mathrm{kJ\ mol^{-1}} \times 2$

(the total energy of step 3 and 4 is –1031 $\mathrm{kJ\ mol^{-1}}$)

5) $\mathrm{Mn^{4+}(g) + 2O^{2-}(g) \rightarrow \varepsilon\text{-}MnO_2(s)}$ $\quad E_{\mathrm{lattice}} = ?\ (-10048.3\ \mathrm{kJ\ mol^{-1}}$

$\mathrm{Mn(s) + O_2(g) \rightarrow \varepsilon\text{-}MnO_2(s)}$ $\quad \Delta G^0_{\mathrm{f}} = -427.3\ \mathrm{kJ\ mol^{-1}}$

In the above cycle, the standard Gibbs energy of sublimation for Mn is assumed to be 238 kJ mol^{-1} (See footnote 2 and ref. [4]; the standard enthalpy of melting and vaporization for Mn are 12.9 and 221 kJ mol^{-1}, respectively from ref. [4]). In addition, the entropy contribution in ionizations are neglected. The ionization potential of Mn^{4+} (ΣI_{p}) and the electron affinity (EA) of O^{2-} are quoted from ref. [6].

2.9 Calculation of the formation Gibbs energy of α-MnOOH and its lattice energy

Since we have determined the standard Gibbs energy of formation for ε−MnO2 and the standard Gibbs energy of the proton insertion/deinsertion reaction, thus the standard Gibbs energy of formation for α-MnOOH can also be calculated as follows:

$$\varepsilon\text{-}\mathrm{MnO_2(s) + \tfrac{1}{2}H_2(g) \rightarrow MnOOH(s)} \qquad \Delta G^0_{\mathrm{rxn}} = -87.5\ \mathrm{kJ\ mol^{-1}} \quad (7)$$

$$\Delta G^0_{\mathrm{rxn}} = \sum \Delta G^0_{\mathrm{f,prod}} - \sum \Delta G^0_{\mathrm{f,react}} = \left[\Delta G^0_{\mathrm{f,MnOOH}}\right] - [-427.3 + 0] = -87.5\ \mathrm{kJ\ mol^{-1}}$$

$$\Delta G^0_{\mathrm{f,MnOOH}} = -514.8\ \mathrm{kJ\ mol^{-1}} \quad (8)$$

Similarly, the calculated standard Gibbs energy of formation can be used in a Born-Haber cycle to calculate the lattice energy of α-MnOOH:

1) $\mathrm{Mn(s) \rightarrow Mn(g)}$ $\quad \Delta G_{\mathrm{sub.}} = 238\ \mathrm{kJ\ mol^{-1}}$

2) $\mathrm{Mn(g) \rightarrow Mn^{3+}(g) + 3e^-}$ $\quad \Sigma I_{\mathrm{p}} = 5496\ \mathrm{kJ\ mol^{-1}}$

3) $\mathrm{\tfrac{1}{2}H_2(g) \rightarrow H(g)}$ $\quad \Delta G_{\mathrm{diss.}} = 436\ \mathrm{kJ\ mol^{-1}} \div 2$

[2] https://www.webelements.com/manganese/thermochemistry.html

4) $H(g) \rightarrow H^+(g)$ $I_p = 1318 \text{ kJ mol}^{-1}$

5) $O_2(g) \rightarrow 2O\ (g)$ $\Delta G_{diss.} = 249 \text{ kJ mol}^{-1}$

6) $2O\ (g) + 4e^- \rightarrow 2O^{2-}(g)$ $EA = -640 \text{ kJ mol}^{-1} \times 2$

(the total energy of step 5 and 6 is –1031 kJ mol^{-1})

7) $Mn^{3+}(g) + 2O^{2-}(g) + H^+(g) \rightarrow MnOOH(s)$ $E_{lattice} = ?\ (-6753.8 \text{ kJ mol}^{-1}$

$Mn(s) + O_2(g) + \frac{1}{2}H_2(g) \rightarrow MnOOH(s)$ $\Delta G_f^0 = -514.8 \text{ kJ mol}^{-1}$

In the above cycle, the ionization potential of H^+ (I_p) and Mn^{3+} ($\sum I_p$) and the electron affinity of O^{2-} (EA) are quoted from ref. [5].

References

[1] D. Han, X. Jing, P. Xu, Y. Ding, J. Liu, *J. Solid State Chem.* **2014**, *218*, 178–183.
[2] M. Musil, B. Choi, A. Tsutsumi, *J. Electrochem. Soc.* **2015**, *162*, A2058–A2065.
[3] C. H. Kim, Z. Akase, L. Zhang, A. H. Heuer, A. E. Newman, P. J. Hughes, *J. Solid State Chem.* **2006**, *179*, 753–774.
[4] J. A. Dean, *Lange's Handbook of Chemistry*, 12th Edition, McGraw-Hill, New York, **1979**.
[5] Y. Marcus, *Ion Properties*, Marcel Dekker, New York, **1997**.
[6] J. Speight, *Lange's Handbook Of Chemistry, Sixteenth Edition*, **2005**.
